# Editorial: Neuro-behavioral insights on low vision and beyond

**DOI:** 10.3389/fnhum.2025.1652389

**Published:** 2025-07-18

**Authors:** Susana Chung, Diego Ghezzi, Ahalya Subramanian, Silvio Ionta

**Affiliations:** ^1^Herbert Wertheim School of Optometry and Vision Science, University of California, Berkeley, Berkeley, CA, United States; ^2^Ophthalmic and Neural Technologies Laboratory, Department of Ophthalmology, Hôpital Ophtalmique Jules-Gonin, University of Lausanne, Fondation Asile des Aveugles, Lausanne, Switzerland; ^3^School of Health and Medical Sciences, City St George's, University of London, London, United Kingdom; ^4^SensoriMotorLab, Department of Ophthalmology-University of Lausanne, Jules Gonin Eye Hospital-Fondation Asile des Aveugles, Lausanne, Switzerland

**Keywords:** vision, rehab, technology, assistance, assessment

The landscape of low vision research is undergoing a profound transformation, driven by the convergence of neuroscience, behavioral science, and technological innovation. This Research Topic offers a panoramic view of how current trajectories are increasingly holistic, patient-centered, and grounded in real-world functionality.

The article by Virgili et al. draws attention to a critical but often overlooked aspect of visual rehabilitation: the burden on informal caregivers. Conducted across four Italian visual rehabilitation centers, the research reveals stress levels indicative of burnout or mental health issues in nearly one out five caregivers' experiences. These findings underscore the urgent need to integrate caregiver support into visual rehabilitation programs.

Grounded in pediatric ophthalmology, the study by Chen et al. shows how innovation is reshaping traditional treatment paradigms. In a randomized controlled trial the authors demonstrate that, compared to conventional patching, gamified binocular vision therapy led to faster initial improvements in visual acuity for children with amblyopia. The engaging nature of the investigated therapy suggests that it could enhance adherence and satisfaction, offering a promising alternative for young patients and their families.

Renton et al. propose a novel approach to study age-related macular degeneration (AMD), combining frequency-tagged videos and electroencephalography to assess spatial frequency processing. Their findings revealed that AMD is associated with reduced sensitivity to high spatial frequencies and increased reliance on low frequencies. The analysis of visual evoked potentials adds support to the theory that AMD may induce cortical reorganization. The combination of neural and behavioral assessments may be a powerful and non-invasive approach for tracking disease progression and tailoring interventions.

The study by Yu et al. focused on driving safety among older adults, an area where traditional visual assessments fall short. Their study examined visual function under various lighting conditions and found that mesopic visual acuity and contrast sensitivity are more predictive of driving ability than standard photopic acuity tests. Using principal component analysis, the researchers identified a minimal set of metrics that explain most of the variance in visual function, providing a foundation for more comprehensive and predictive assessments of driving fitness in aging populations.

In the realm of assistive technology, Nejad et al. investigated a deep learning model for simulated prosthetic vision (Point-SPV). By incorporating gaze-based optimization and task-specific training, the model significantly improved object recognition performance. This research exemplifies how artificial intelligence can be harnessed to align prosthetic outputs with real-world visual demands, enhancing both functionality and user experience.

Swain et al.'s study conducted in rural environments sheds light on the quality of life among patients at risk for glaucoma. They found that factors such as driving status, insurance coverage, and visual symptoms significantly influenced vision-related quality of life. These findings highlight the importance of accessible, culturally sensitive screening tools and support the nine-item National Eye Institute Visual Function Questionnaire (NEI VFQ-9) as a valuable outcome measure for evaluating interventions in underserved communities.

With a focus on cerebral visual impairment in children, Chandna et al. introduced the Higher Visual Function Deficit spectrum and severity indices and validated the Higher Visual Function Question Inventory. Their study demonstrated that higher visual function deficits can occur independently of acuity loss. This paradigm shift advocates for a more nuanced, neurodevelopmental model of pediatric visual assessment, with implications for both clinical practice and educational support.

Altogether, this Research Topic illustrates paradigm shifts in low vision research, from a narrow focus on acuity to a broader understanding of functional vision, neural adaptation, and psychosocial context. As we move forward, the integration of advanced diagnostics, personalized therapies, and inclusive care models will be essential to improving outcomes for individuals with visual impairments across the lifespan.

